# Design of a Virtual Player for Joint Improvisation with Humans in the Mirror Game

**DOI:** 10.1371/journal.pone.0154361

**Published:** 2016-04-28

**Authors:** Chao Zhai, Francesco Alderisio, Piotr Słowiński, Krasimira Tsaneva-Atanasova, Mario di Bernardo

**Affiliations:** 1 Department of Engineering Mathematics, University of Bristol, BS8 1UB Bristol, United Kingdom; 2 College of Engineering, Mathematics and Physical Sciences, University of Exeter, EX4 4QF Exeter, United Kingdom; 3 Department of Electrical Engineering and Information Technology, University of Naples Federico II, 80125 Naples, Italy; Champalimaud Foundation, PORTUGAL

## Abstract

Joint improvisation is often observed among humans performing joint action tasks. Exploring the underlying cognitive and neural mechanisms behind the emergence of joint improvisation is an open research challenge. This paper investigates jointly improvised movements between two participants in the mirror game, a paradigmatic joint task example. First, experiments involving movement coordination of different dyads of human players are performed in order to build a human benchmark. No designation of leader and follower is given beforehand. We find that joint improvisation is characterized by the lack of a leader and high levels of movement synchronization. Then, a theoretical model is proposed to capture some features of their interaction, and a set of experiments is carried out to test and validate the model ability to reproduce the experimental observations. Furthermore, the model is used to drive a computer avatar able to successfully improvise joint motion with a human participant in real time. Finally, a convergence analysis of the proposed model is carried out to confirm its ability to reproduce joint movements between the participants.

## Introduction

Human social interactions give rise to a variety of self-organized and emergent motor behaviors [1–4]. A typical example is joint improvisation between two humans performing some task together, as engaging in a conversation or public performance [5–7]. Experimental results suggest that coordination in joint actions unconsciously fosters social rapport and promotes a sense of affinity between two individuals [8, 9].

To investigate the mechanisms behind the emergence of social interaction between two individuals, the Human Dynamic Clamp paradigm has been recently proposed in [4, 10, 11] where a model-driven avatar (or virtual player) replaces one of the two humans. In so doing, the features of the motion of the virtual player (VP) can be manipulated in order to understand whether and how the interaction with the human subject is affected.

Here we present a mathematical framework for the study of joint improvisation between two participants, to whom no roles of leader and follower are assigned beforehand. In particular, we first propose a set of metrics to quantify some features of joint improvisation. Then, taking a top-down approach in which we make some hypotheses on the key factors governing joint improvisation as defined by such metrics, we propose a mathematical model able to reproduce *in-silico* the results observed experimentally from the interaction between two humans. We also use such mathematical description, based on optimal control theory, to control a model-driven virtual player and enable it not only to interact with a human subject, but also to generate jointly improvised movements with him/her.

We focus on the mirror game, a paradigm of joint human interaction which was recently proposed in [12, 13] (see [14] for more details on the link between mirror game and psychological constructs of attachment). Contrary to previous approaches where models were typically used to generate simple oscillatory motion or to reproduce the motion of a human subject [4], we present a model able to capture the complex movements generated by human subjects playing the game and generate new motion. Furthermore, we show that the model can capture and reproduce the essential kinematic features of the movement of a reference human subject as encoded by the Individual Motor Signature recently introduced in [15, 16], opening the possibility of testing *in-silico* the interaction between different individuals in a number of different configurations.

The ability of the model to reproduce the experimental results and its ability to drive a computer avatar in real-time are tested and validated via an extensive numerical investigation, accompanied by a mathematical analysis of its convergence.

## Materials and Methods

### Mirror game

**Fig 1:**
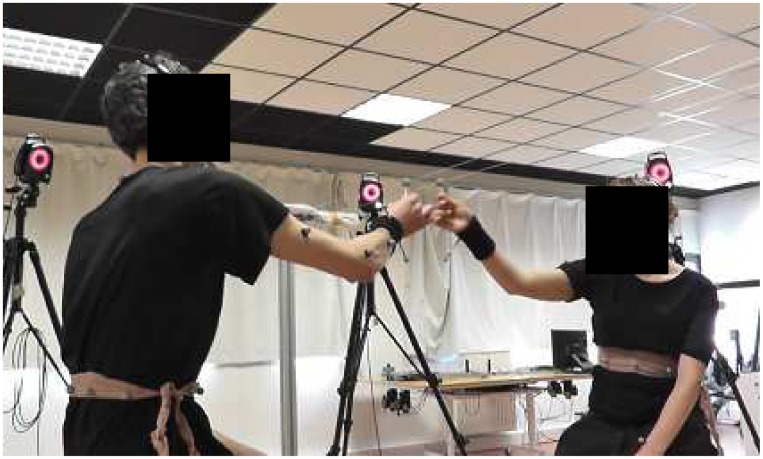
Mirror game between two human players at University of Montpellier, France. Two participants face each other and are asked to perform synchronized motion by moving two balls along a string to which they are respectively attached.

As a simple yet effective paradigm to study interpersonal coordination between two individuals we use the mirror game [12]. Specifically, as shown in Fig 1, two players facing each other are asked to coordinate the motion of two balls mounted on two respective parallel strings. The players can be asked to play in a leader-follower condition (LF), where one is instructed to follow the motion of the other, or in a joint improvisation condition (JI), where they are instructed to imitate each other, create synchronized and interesting motions and enjoy playing together, without any designation of leader and follower. Only the latter experimental condition will be considered in this paper.

All the study discussed in this work was carried out according to the principles expressed in the Declaration of Helsinki and approved by the local ethical committee (University of Montpellier, France). The participants provided verbal informed consent to participate in the study, and such consent was approved by the ethics committee. Written consent was not necessary given the small pre-test nature of the experiments and full anonymization of the data.

### Individual motor signature

As shown in [15, 16], the motion of each individual in the mirror game is characterized by different kinematic features that can be accounted for by examining the velocity profile of the player’s motion during the game. The velocity profile comprises a velocity frequency distribution, termed in [16] as Individual Motor Signature (IMS), and can be used to classify and distinguish the movement generated by different human subjects. To acquire the IMS of a human subject, we asked him/her to play the mirror game in a Solo condition, i.e. in the absence of the other participant. In this condition, the human subject was asked to generate interesting complex motion for 60 seconds. The position time series were recorded during the experiment, and were next used to estimate the velocity PDF of the player’s motion.

### Experimental set-ups

We use two experimental set-ups to carry out experiments.

**Fig 2:**
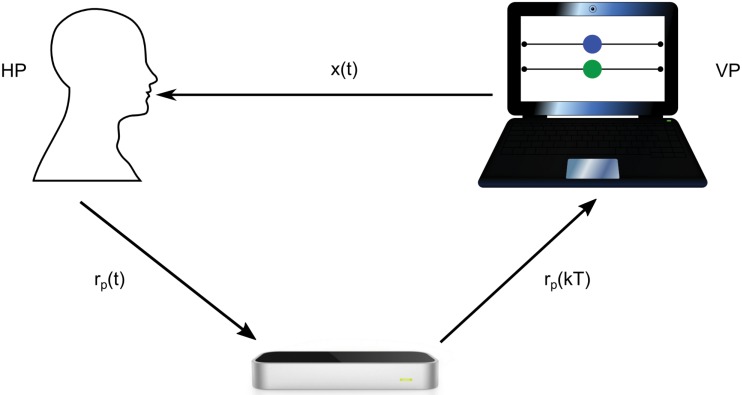
Experimental setup of the mirror game between a HP and a VP. The position of the human fingertip *r*_*p*_(*t*) is detected by a leap motion controller, and the sampled position *r*_*p*_(*kT*) is sent to the computer, while the position *x*(*t*) of the VP is generated by implementing the numerical algorithm of the single model. Two circles are shown on the computer screen, which correspond to the end effectors’ position of the HP (blue circle) and the VP (green circle), respectively.

- Set-up 1 (shown in Fig 1) consists of two parallel strings (180 cm long), with a ball that can slide on each. Two human players are asked to move their own ball back and forth along the strings, respectively, while seated. The position of the balls are detected by cameras disposed around the two participants. Details of the set-up can be found in [15, 16].
- Set-up 2 (schematically shown in Fig 2) employs a cheap leap motion controller, whose spatial accuracy is below 0.2*mm* [17], in order to detect the fingertip position of the human player (HP).
  Both the leap motion controller and a laptop computer (employed to implement and run the theoretical model driving the computer avatar) are placed on a table. The HP is asked to wave his/her own index finger horizontally over the leap motion controller with a horizontal range of around 60*cm*. The fingertip position of the HP is mapped into the interval [−0.5, 0.5] and visualized as a blue solid circle on the computer screen. A green solid circle, whose movement is computed from the model presented in this paper, is also visualized to represent the position of the virtual player. The advantage of using this simple set-up, consisting of cheap off-the-shelf elements, is its accessibility and ease of implementation.

It is worth pointing out that, due to the longer range of movement in experimental set-up 1, the generated position trace is affected mostly by the motion of arm, forearm and palm, while in experimental set-up 2 the movement generation involves mostly forearm and palm. As a consequence, the motion captured by means of experimental set-up 2 exhibits generally a simpler trajectory as the end-effector system has less degrees of freedom. Nevertheless, as shown in [16], both set-ups can be used to capture individual characteristics of motion of different participants, and hence be treated as equivalent.

### Data and evaluation metrics

In order to assess the level of coordination between the players and define some of the typical features of joint improvisation in the mirror game, we use the following metrics.

- *Position temporal correspondence*. The root mean square (RMS) of the normalized position error between the two players defined as
  $$e_{p}\;=\;\frac{1}{L}\sqrt{\frac{1}{n}\;\sum_{k=1}^{n}{\left(x_{1,k}-x_{2,k}\right)}^{2}}$$
  is used to describe their movement synchronization during the game. Here *L* refers to the range of admissible positions (e.g. the length of the strings in the set-up shown in Fig 1 or the range of motion detected by the leap motion controller), *n* is the number of sampling steps in the simulation, and *x*_1,*k*_ and *x*_2,*k*_ denote the positions of two participants at the *k*-th sampling step, respectively.
- *Distance between IMSs*. The earth mover’s distance (EMD) is used to quantify differences between the velocity PDFs of the two players’ motion (e.g. to assess how similar/dissimilar their IMSs are). It is a proper metric in the space of PDFs [16, 18] and is computed as follows:
  $$\eta \left(p_{1},p_{2}\right)=\int_{Z}\left|CDF_{p_{1}}\left(z\right)-CDF_{p_{2}}\left(z\right)\right|dz$$
  where *Z* denotes the integration domain, and *CDF*_*p*_*i*__(*z*) denotes the cumulative distribution function of the distribution *p*_*i*_, *i* ∈ {1, 2}. Furthermore, we normalize the EMDs with the maximal *η*_*max*_ given by the length of the integration domain *η*_*max*_ = |*Z*|.
- *Relative phase distribution*. The PDF of the relative phase *ϕ*_12_ between the players motion (estimated by means of wavelet coherence [19]) is used to check the directionality of the interaction during the game (i.e. if one player is leading or following the other).

### Human benchmark

**Fig 3:**
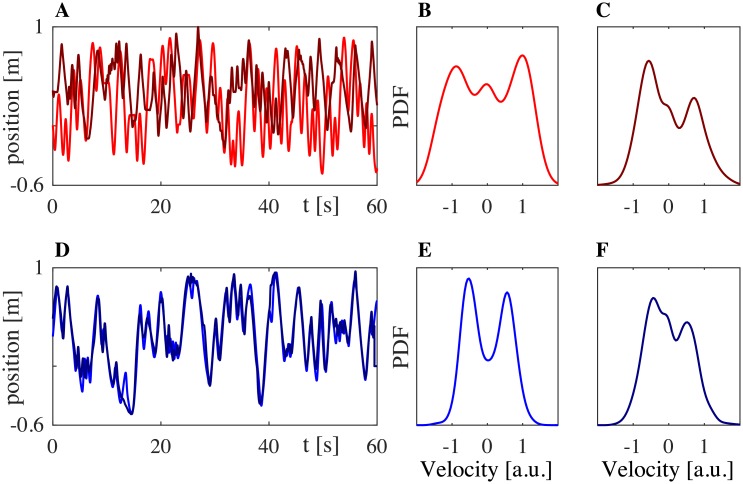
Example of position time series and velocity PDFs from experimental results for Dyad 1. A: position time series for the solo trials of the HPs. B-C: PDFs of velocity corresponding to the two players. D: position time-series of the HPs from the JI trial. E-F: PDFs of velocity of the two HPs in JI condition.

To establish a benchmark dataset to compare with the model simulations, we obtained data from 8 different human dyads. Data from each dyad contains 3 solo trials for each human participant and 3 joint trials between them in JI condition. Description of all the available data (Matlab structure in S1 Dataset) can be found in Section B of S1 File. A representative example of the data collected in the mirror game between two HPs (Dyad 1, HPs JI trial 3) is shown in Fig 3. Here below we list the main observations that guided us towards a definition of joint improvisation.

**Fig 4:**
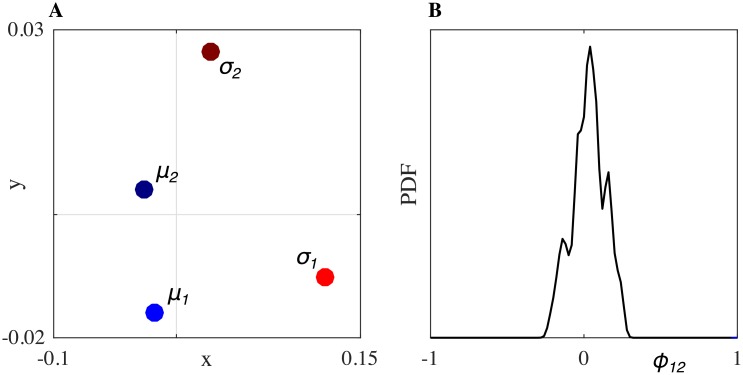
Example of data analysis for Dyad 1. A: visualization of the relations between kinematics of two human players obtained by means of MDS. Red dots *σ*_1,2_ indicate the signatures of the two respective HPs. Blue dots labelled as *μ*_1,2_ indicate the velocity profile of the motion of the HPs in the JI trial. B: PDF of relative phase *ϕ*_12_ between the two HPs.

**Fig 5:**
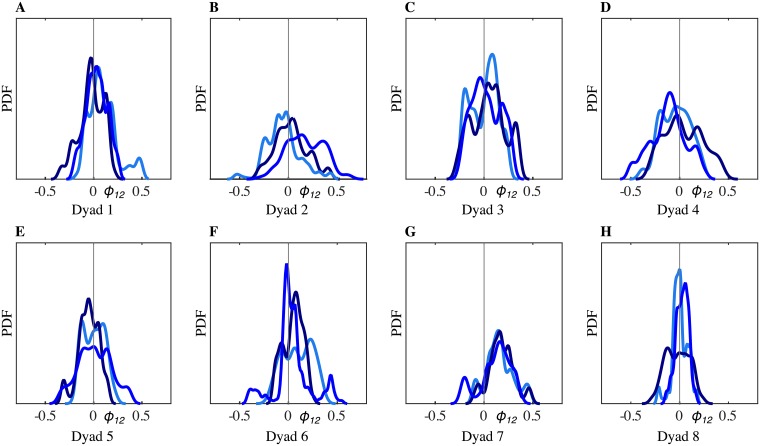
PDFs of relative phase between HPs in all the dyads. Each panel corresponds to a single dyad. For each dyad, three HP_1_-HP_2_ trials are shown with different scales of blue. The relative phase *ϕ*_12_ between players is estimated by means of wavelet coherence (with 1Hz cut-off frequency). PDFs are estimated from histograms with a kernel density estimation.

1. Fig 3A shows the trajectories of the first solo trial of HP_1_ (light red) and the third solo trial of HP_2_ (dark red), while Fig 3D shows the trajectories of the two HPs interacting in JI condition (light and dark blue). Additionally, we show the velocity PDFs estimated from each of the respective position time series: panels B-C from Solo and panels E-F from JI between the two HPs. The low value of RMS position error observed experimentally (*e*_*p*_ ≃ 0.08) shows that the two players managed to reach a good level of movement synchronization when interacting together.
2. In order to visualize the relations between the distributions, we used multidimensional scaling (MDS), a data mining and visualization technique [16, 20] that uses distances/ dissimilarities between objects to represent them as points in a geometric space. An example of such a geometric representation of the relations between the PDFs of the players’ velocities is shown in Fig 4A. Different velocity PDFs are denoted with different markers: *σ*_*i*_ (red dots) indicates the motor signature of the *i*-th human player when playing solo; *μ*_*i*_ (blue dots) indicates the velocity PDFs of the *i*-th human player during runs of the mirror game with the other player.
  In agreement with previous studies [13], we found that the kinematic characteristics of motion change with respect to solo conditions when the participants are improvising together. Namely, velocity PDFs of the two HPs (Fig 4A, light and dark blue dots) move away from their respective motor signatures (Fig 4A, light and dark red dots), because of mutual imitation, adaptation and synchronization, which results in their velocity PDFs moving towards each other during the game. In other words, the players exhibit behavioral plasticity as defined in [16]. The values of the distances between the velocity PDFs depicted in Fig 4A were computed to be *η*(*σ*_1_, *μ*_1_) = 0.102, *η*(*σ*_2_, *μ*_2_) = 0.052 and *η*(*μ*_1_, *μ*_2_) = 0.030.
3. Finally, in Fig 4B, we plot the distribution of the relative phase between the two players. We found that it is quite broad and centered around 0, indicating that neither of the two players was clearly leading the interaction during the game.
  Similar results were obtained for all the trials of every dyad (Fig 5). Each panel of Fig 5 corresponds to a single dyad. For each dyad, three PDFs of relative phase from the three respective JI trials are shown with different scales of blue. It is possible to appreciate how all the PDFs are quite broad and centered around 0. The only exception is Dyad 7 where the maximum of the relative phase PDF is shifted on the right for all the three trials indicating that, despite the instruction given to the two participants, one player consistently ended up leading the game (Fig 5G).

## Results

### Theoretical model of joint improvisation

**Fig 6:**
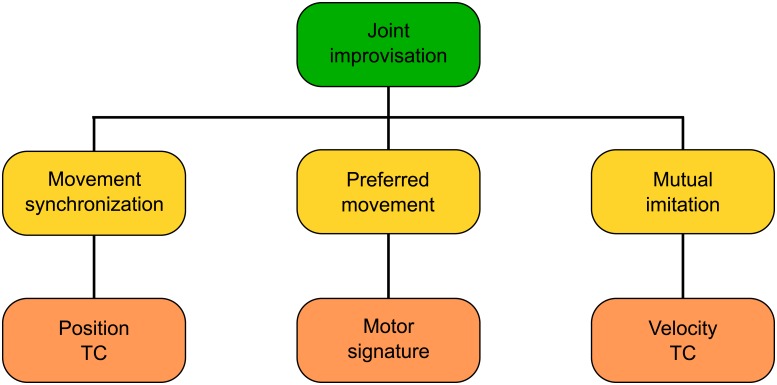
Theoretical model of joint improvisation. Three key factors governing JI (from left to right): 1. movement synchronization, corresponding to temporal correspondence (TC) between positions of the players’ end effectors; 2. preferred movement, captured by their respective IMS; 3. mutual imitation, modeled by temporal correspondence between their velocities.

Our first result is a mathematical model of Joint Improvisation. Our experimental observations of two HPs playing the mirror game suggest that their interaction in a JI condition is driven by three key factors: (i) their will to synchronize each other’s movement; (ii) the tendency of each player to exhibit some individual preferred movement features (or IMS); and (iii) the attempt each player makes to imitate the way the other moves (or mutual imitation). As shown in Fig 6, we proposed to map each of these three factors onto a specific behavioral goal. In particular, *synchronization of joint movements* can be translated into the goal of minimizing the position mismatch between the balls moved by the two participants, which is related to the temporal correspondence (TC) between their positions. *Spontaneous motion* preferences arise from the tendency of each participant to move according to his/her own IMS. Finally, *mutual imitation* can be achieved by the participants minimizing their velocity mismatch (velocity TC) during the mirror game. We captured each of these properties into a new mathematical model of interaction during JI formulated as the following optimal control problem.

Specifically, following the approach of [21–24], we modeled the motion of each of the players using a nonlinear Haken-Kelso-Bunz (HKB) oscillator [25] of the form

$$\begin{array}{c} \ddot{x_{i}}+\left(\alpha_{i}{\dot{x_{i}}}^{2}+\beta_{i}x_{i}^{2}-\gamma_{i}\right)\dot{x_{i}}+\omega_{i}^{2}x_{i}=u_{i},\;i=1,2 \end{array}$$ (1)

where *x*_*i*_ and ${\dot{x}}_{i}$ denote position and velocity of player *i*, *u*_*i*_ is the coupling function through which player *i* modulates its motion according to that of the other player, while *α*_*i*_, *β*_*i*_, *γ*_*i*_ and *ω*_*i*_ are intrinsic parameters determining the intrinsic properties of the player’s motion, such as speed of reaction and settling time. We represented the coupling function *u*_*i*_ as a nonliner control input that each player computes by minimizing the following cost function over each sampling period *T* = *t*_*k*+1_ − *t*_*k*_ the whole trial duration is being split into. Namely, the problem is that of finding the inputs *u*_*i*_ such that

$$\begin{array}{c} \min_{u_{i}\in R}\;J_{i}\left(x_{1},x_{2},{\dot{x}}_{1},{\dot{x}}_{2},t\right) \end{array}$$ (2)

where

$$\begin{array}{c} \begin{array}{cc} J_{i}\left(x_{1},x_{2},{\dot{x}}_{1},{\dot{x}}_{2},t\right) & =\frac{\theta_{p,i}}{2}\underset{Position\;TC}{\underset{︸}{{\left(x_{1}\left(t_{k+1}\right)-x_{2}\left(t_{k+1}\right)\right)}^{2}}}+\frac{\theta_{\sigma ,i}}{2}\int_{t_{k}}^{t_{k+1}}\underset{Motor\;Signature}{\underset{︸}{{\left({\dot{x}}_{i}\left(\tau \right)-\sigma_{i}\left(\tau \right)\right)}^{2}}}d\tau \\ & +\frac{\theta_{v,i}}{2}\int_{t_{k}}^{t_{k+1}}\underset{Velocity\;TC}{\underset{︸}{{\left({\dot{x}}_{1}\left(\tau \right)-{\dot{x}}_{2}\left(\tau \right)\right)}^{2}}}d\tau +\frac{\eta_{i}}{2}\int_{t_{k}}^{t_{k+1}}u_{i}{\left(\tau \right)}^{2}d\tau \end{array} \end{array}$$ (3)

with *θ*_*p*,*i*_, *θ*_*σ*,*i*_, *θ*_*v*,*i*_, *η*_*i*_ > 0 being tunable control parameters satisfying the constraint *θ*_*p*,*i*_ + *θ*_*σ*,*i*_ + *θ*_*v*,*i*_ = 1. Here, *σ*_*i*_ encodes the IMS of player *i* as his/her velocity time series during solo trials.

The cost function described in Eq (3), which is a more general form of that proposed in [22–24] as it includes the leader-follower model, contains four terms. The first three terms correspond to each of the three factors characterizing JI shown in Fig 6, while the fourth aims at minimizing the control effort over each sampling period. Indeed, the three tunable weights *θ*_*p*,*i*_, *θ*_*σ*,*i*_ and *θ*_*v*,*i*_ allow for movement synchronization (position TC), preferred movement (motor signature) and mutual imitation (velocity TC), respectively, while *η*_*i*_ allows to regulate the control energy, i.e. the amplitude of the control signal *u*_*i*_. Different set values for *θ*_*p*,*i*_, *θ*_*σ*,*i*_ and *θ*_*v*,*i*_ can be used to change the balance between the terms described above. In the most trivial cases:

- setting *θ*_*p*_ = 1, *θ*_*σ*_ = 0 and *θ*_*v*_ = 0 would make the VP behave as a perfect follower, as it would simply reproduce the movement of the other player;
- setting *θ*_*p*_ = 0, *θ*_*σ*_ = 1 and *θ*_*v*_ = 0 would make the VP behave as a blind leader, as it would simply replay a pre-recorded trajectory without taking into account the behavior of the other player;
- setting *θ*_*p*_ = 0, *θ*_*σ*_ = 0 and *θ*_*v*_ = 1 would make the VP copy the velocity of the other player without considering the position mismatch (a finite sampling rate might lead to a large shift between the positions of the two players).

In other words, the optimal control framework allows to incorporate into the same cost function all the key factors identified to govern joint improvisation. In what follows we will refer to each of the players modelled by Eqs from (1) to (3) as a *virtual player*. We will denote VPs as VP_*i*_ where *i* denotes that the model receives as an input the pre-recorded velocity profile *σ*_*i*_ of the *i*-th HP playing solo.

### Model testing and validation

**Fig 7:**
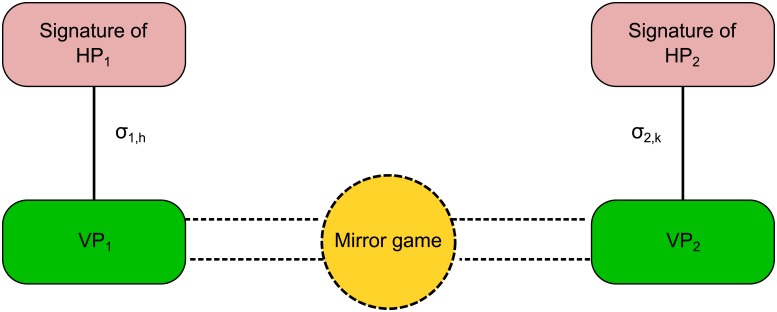
Schematic diagram of VP-VP interaction. VP_1_ is fed with the motor signature of HP_1_, while VP_2_ with that of HP_2_. We refer to the IMS of the *i*-th HP recorded in the *j*-th Solo trial of each dyad as *σ*_*i*,*j*_. In this case *h*,*k* = 1,2,3 give rise to 9 different combinations for each dyad. The JI session played by the virtual players resembles the one performed by the two HPs, whose respective motor signatures are fed to the VPs.

To test the effectiveness of the model, we compared numerical results with the human benchmark described in Section *Human benchmark*. A total of 9 different interactions in each of the 8 dyads were considered, since there were 3 solo trials available for each participant (corresponding to the reference velocity profiles *σ*_*i*_ used in the model). Indeed, if we refer to the IMS of the *i*-th HP recorded in the *j*-th Solo trial of each dyad as *σ*_*i*,*j*_, the 9 different interactions were obtained by feeding VP_1_ with *σ*_1,*h*_ and VP_2_ with *σ*_2,*k*_, where *h*,*k* = 1,2,3 (Fig 7). Description of all the available data (Matlab structure in S2 Dataset) and more information on the composition of all dyads can be found in Section B of S1 File.

In so doing, for each dyad, each model equation was used to describe the kinematic behavior of a corresponding HP. The parameters of the model were set heuristically to the following values: *α*_1_ = *α*_2_ = 1, *β*_1_ = *β*_2_ = 1, *γ*_1_ = *γ*_2_ = 1, *ω*_1_ = *ω*_2_ = 1, *T* = *t*_*k*+1_−*t*_*k*_ = 0.016s. The weights *θ*_*p*,*i*_, *θ*_*σ*,*i*_ and *θ*_*v*,*i*_ were also set heuristically by trial-and-error in order to best match the experimental results (see Table A in Section A of S1 File for further details on the values of the weights and how to interpret them).

#### Single JI trial comparison

For the sake of clarity, we begin by showing a quantitative comparison of experimental data from a single HP-HP dyad with the corresponding data obtained by simulation of the model equations. In particular, we considered the third JI trial of Dyad 1. We denote by *ν*_*i*_ the velocity PDF of VP_*i*_ and by *μ*_*i*_ the velocity PDF of its corresponding HP_*i*_ from a simulated and experimental JI trial, respectively.

The values of the EMDs between velocity PDFs are given in Table 1. We found that the distance *η*(*σ*_*i*_, *ν*_*i*_) between the velocity PDFs of each virtual player (evaluated from the JI trial) and its reference motor signature *σ*_*i*_ matches closely the distance *η*(*σ*_*i*_, *μ*_*i*_) between the corresponding HPs and their own signatures. Moreover, the distance *η*(*ν*_*i*_, *ν*_*j*_) between two VPs interacting with each other is quite close to that observed when the two HPs they model interact together in the mirror game, *η*(*μ*_*i*_, *μ*_*j*_). This shows how, just like in the case of two humans (previously analyzed in Section *Human benchmark*), the velocity PDFs of the two VPs move away from their respective signatures and get close to each other (while remaining close to the velocity PDFs of their human counterparts in the JI trial).

**Table 1:** Evaluation of the model via EMDs between velocity PDFs for a single trial.

Motor signature		Interaction	
*η*(*σ*_1_, *μ*_1_)	0.102	*η*(*μ*_1_, *μ*_2_)	0.030
*η*(*σ*_1_, *ν*_1_)	0.142	*η*(*ν*_1_, *ν*_2_)	0.021
*η*(*σ*_2_, *μ*_2_)	0.052	*η*(*ν*_1_, *μ*_1_)	0.052
*η*(*σ*_2_, *ν*_2_)	0.067	*η*(*ν*_2_, *μ*_2_)	0.019

Here *μ*_*i*_ denotes the velocity profile of HP_*i*_ during an experimental interaction with the other human player, *ν*_*i*_ that of the corresponding VP_*i*_ playing with another VP *in-silico*, and *σ*_*i*_ are the pre-recorded IMSs of the HPs.

Computation of the relative phases PDFs in HP-HP interactions and VP-VP interactions confirmed that they are close to each other, thus leading to the conclusion that, just like in the human scenario, neither of the two VPs turned out to be a leader during the JI trial. Indeed, if we denote with *ϕ*_*VP*_ and *ϕ*_*HP*_ the PDFs of the relative phase between the two VPs and the two HPs they model, respectively, the EMD between them was computed to be *η*(*ϕ*_*VP*_, *ϕ*_*HP*_) = 0.024.

The previous findings show how the proposed model succeeds in capturing the main characteristics of the interaction between two human players improvising together, thus demonstrating its ability to reproduce *in-silico* the mirror game between two human subjects in a JI condition.

#### Matching results for the 8 dyads

Next, we present results for all the 8 experimental data-sets and show how our model is able to capture the experimental observations in terms of: *1)* RMS position error, *2)* changes in EMD between velocity PDFs and *3)* EMD between relative phase PDFs.

**Fig 8:**
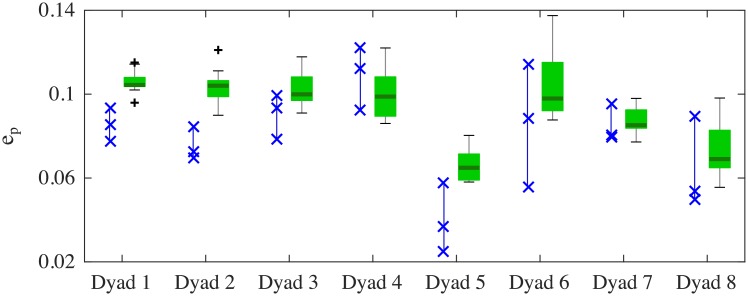
Matching in terms of position temporal correspondence between HPs and between VPs. Blue crosses (x) show the RMS position error from three JI trials of HPs, whilst box-plots depict the distributions of RMS position error from nine simulations of JI interaction between VPs (corresponding to nine combinations of the HPs’ individual signatures). In particular: thick horizontal green lines indicate median of the distribution; central light green boxes show central 50% of the data with lower and upper boundary lines being at the 25% and 75% quantiles of the data; two vertical whiskers estimate 99% of the range of the data; black crosses (+) show outliers.

**Fig 9:**
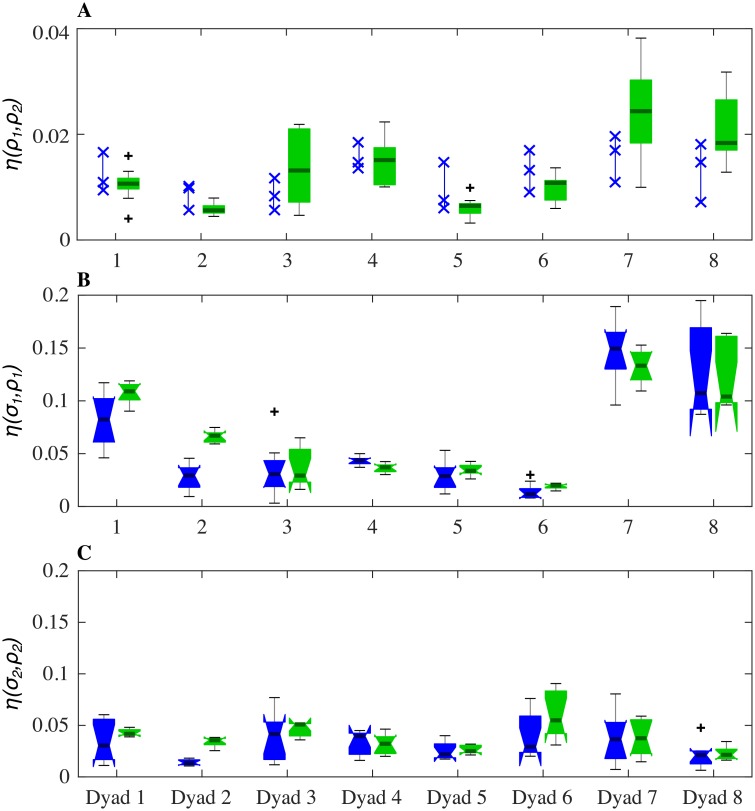
Matching in terms of relations between kinematics of HPs and VPs. *ρ*_*i*_ denotes the velocity PDF of the *i*-th player from a JI trial, that is *μ*_*i*_ for HP_*i*_ and *ν*_*i*_ for VP_*i*_. A: degree of similarity between PDFs of velocities recorded during JI trials. Blue crosses show 3 trials for HPs and the green box-plots depict distributions of EMDs between velocities from 9 JI trials between VPs. B and C show how far the movements of the players in JI condition were shifted away from their motor signatures (blue for HPs, green for VPs). Notches on the box-plots indicate confidence intervals of the medians. Two medians are significantly different at the *p* = 0.05 level if their notches do not overlap. Notches extending beyond the box indicate that the confidence intervals extend beyond central 50% of the data points.

**Fig 10:**
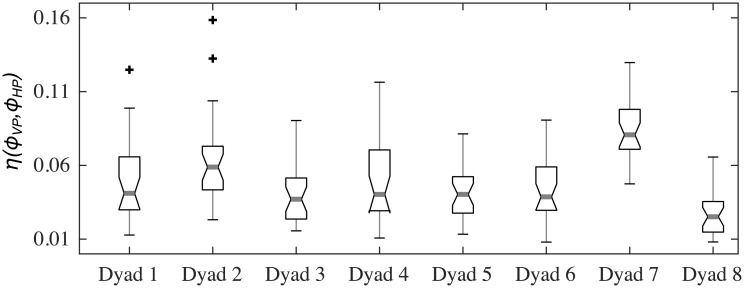
Matching in terms of relative phase between HPs and between VPs. Box-plots illustrate distributions of EMDs between PDFs of relative phase from JI trials of HPs and VPs. Each box-plot corresponds to a single dyad and is constructed from 27 EMDs between three HP-HP relative phase PDFs and nine for the coupled VPs; black crosses (+) show outliers.

1. Fig 8 shows good agreement between RMS position errors observed in the experiments (blue crosses, three trials) and those obtained from corresponding VP dyads (green boxes, nine trials). In particular, it is worth pointing out that good levels of movement synchronization are achieved for both HP-HP and VP-VP dyads (*e*_*p*_ is always below 14%), and how the higher level of synchronization between HPs in Dyad 5 (lower value of *e*_*p*_) was also captured in the VP-VP simulations.
2. Fig 9 confirms the model ability to capture the behavioral plasticity of the IMS of the players during a typical game. Data from JI trials between HPs is shown in blue, while data corresponding to the VPs simulations is shown in green. We found that the EMD between velocity PDFs of two HPs was similar to that of the two corresponding VPs for all the dyads (Fig 9A). Moreover, such similarity was found also in the relations between each player’s motion and their signatures (Fig 9B and 9C). It is worth pointing out how the higher value of *EMD* between HP_1_ and his/her corresponding signature for Dyad 7 and Dyad 8 was well captured by the model simulations (Fig 9B).
3. Fig 10 shows a quantitative comparison of the PDFs of the relative phase computed from trials between HPs and VPs dyads. It is possible to appreciate how the EMD between relative phases in VP-VP trials, *ϕ*_*VP*_, and HP-HP trials, *ϕ*_*HP*_, is low for all the eight dyads. This means that the absence of an emerging leader observed in a JI session between humans was also replicated in simulations between corresponding virtual players.

### A model-driven avatar

We used our theoretical model to drive a computer avatar able to play the mirror game with a human player in a JI condition, such that the features previously analyzed for human dyads could be reproduced in HP-VP interactions as well. We employed the low-cost experimental set-up 2 described in Section *Experimental set-ups* where the human player moves one of the two solid circles on the screen via a leap motion controller while the other is moved by the computer avatar. Description of all the available data (Matlab structure in S3 Dataset) can be found in Section B of S1 File.

The avatar computes the position of the circle it is moving by solving just one of Eq (1), say for *i* = 1, with *u*_1_ obtained by solving the optimal control problem described in Eq (3). Now, *x*_2_ and ${\dot{x}}_{2}$ indicate position and velocity of the HP (whose signature is denoted with *σ*_*HP*_) interacting with the VP. In particular, velocity and position of such human player are estimated over each interval according to

$$\begin{array}{c} {\dot{x}}_{2}\left(t\right)=\frac{x_{2}\left(t_{k}\right)-x_{2}\left(t_{k-1}\right)}{T}\;t\in \left[t_{k},t_{k+1}\right] \end{array}$$

and

$$\begin{array}{c} x_{2}\left(t\right)=x_{2}\left(t_{k}\right)+{\dot{x}}_{2}\left(t\right)\left(t-t_{k}\right),\;t\in \left[t_{k},t_{k+1}\right] \end{array}$$

Moreover, *σ*_1_ in Eq (3) indicates the IMS of a different HP (which is fed to the VP) and is denoted with *σ*_*VP*_.

All the other model parameters were selected heuristically as follows: *α*_1_ = 1, *β*_1_ = 1, *γ*_1_ = 1, *ω*_1_ = 1, *η*_1_ = 10^−4^, *T* = *t*_*k*+1_−*t*_*k*_ = 0.04s, *θ*_*p*,1_ = 0.2, *θ*_*σ*,1_ = 0.4 and *θ*_*v*,1_ = 0.4. The initial position and velocity of the avatar were set to 0.

**Fig 11:**
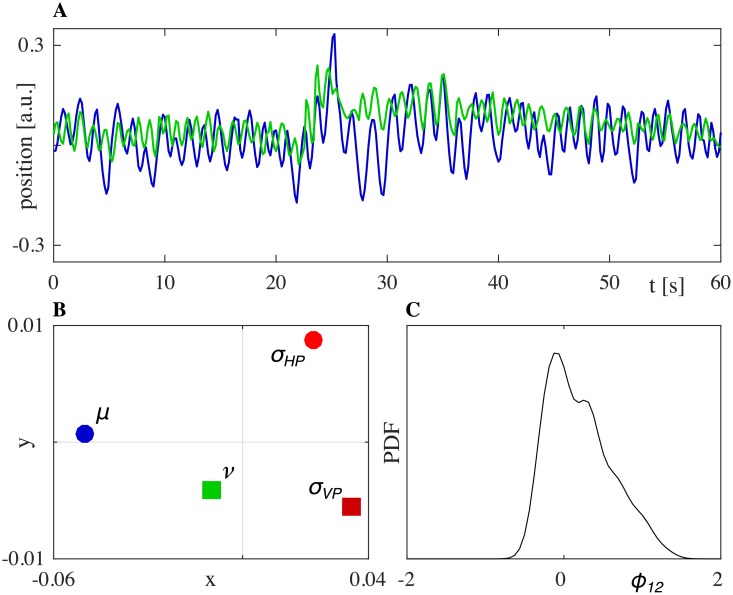
Interaction between a VP and a HP. A: positions of HP (blue) and VP (green). B: relations between kinematics of motion of the players visualized by means of MDS. C: PDF of the relative phase between the two time-series of A.

The position time series recorded in the experiment for both players are shown in Fig 11A. We remind the reader that the different visual appearance of the position traces in Figs 3D and 11A results from the differences in the experimental set-ups. However, we would like to emphasize that both illustrated interactions satisfy our description of JI. More specifically, we found that the main features of JI observed in the case of HP-HP interaction were replicated when replacing one of the two HPs with a VP. Indeed:

1. the value of the RMS of the normalized position error between HP and VP (*e*_*p*_ ≃ 0.16) is comparable with that obtained in HP-HP interactions (Fig 8), showing that in both cases a good level of movement synchronization is achieved;
2. both HP and VP move away from their own signatures and converge towards each other (Fig 11B). In particular, *η*(*ν*, *σ*_*VP*_) = 0.048, *η*(*μ*, *σ*_*HP*_) = 0.074 and *η*(*μ*, *ν*) = 0.042, with *μ* and *ν* being the velocity PDFs obtained from the JI interaction between HP and VP, respectively;
3. the wide PDF of the relative phase between HP and VP indicates that there is no effective leader during the interaction (Fig 11C).

Both results confirm that a computer avatar driven by our theoretical model is able to jointly improvise its motion in real-time with a human subject providing a new powerful tool for discovery and investigation of social interaction and movement coordination in the mirror game.

### Convergence analysis

Finally, we confirmed via a theoretical analysis that the model we propose guarantees convergence between the players when either two coupled VPs are considered or when the model-driven avatar interacts with a human subject. Our main stability results can be listed as follows (see S1 Appendix for a proof of the findings and further details):

- the solution to the minimization problem described from Eqs (1) to (3) ensures bounded position error between two VPs when *in-silico* experiments are considered;
- the solution to the minimization problem described from Eqs (1) to (3) ensures bounded position error between HP and VP when the model-driven avatar interacts with a human subject;
- if the nonlinear HKB dynamics of the VP end-effector described in Eq (1) is substituted with a simpler linear model, achievement of the optimal solution to the minimization problem described in Eqs (2) and (3) is guaranteed over each subinterval.

## Discussion

In this work we presented a new mathematical model, based on the use of a nonlinear oscillator and an optimal control theoretic framework, to explain and reproduce joint improvisation in human dyads as defined by the introduction of appropriate metrics. Using both model simulations and experiments, we demonstrated the applicability of our modelling approach to capture the features of joint improvisation between two human players in the mirror game, and its capability to drive a computer avatar to produce jointly improvised movements with a human player, respectively. Indeed, both VP-VP and HP-VP interactions exhibited the main characteristics of JI as defined within the context of HP-HP interactions in the mirror game. Specifically: *1)* high levels of movement synchronization, by means of a low value for the RMS of the normalized position error between the agents; *2)* behavioral plasticity of the players, measured by means of changes in EMD between velocity profiles; *3)* absence of a clear leader, by means of a relative phase distribution of the players centered around 0.

The availability of such an enhanced model-driven avatar provides a new fundamental tool to explore the important tenet in Social Psychology that behavioral similarity between people facilitates their interaction [26, 27]. In particular, as recently proposed in [16], the similarity or dissimilarity between the IMSs of two individuals playing the mirror game can be an important factor affecting the level of their mutual interaction and coordination. Our proposed model can be used to test this hypothesis both *in-silico* and via real-time experiments.

Future work will include finding a strategy that would help to appropriately choose the weights in the cost function. One approach could be to implement adaptive laws to make the weights vary over time during the game session, according to the performance evaluated in real time. Another possible extension of our work includes the possibility of carrying out Turing-test experiments, where human participants are asked to perform joint improvisation with another agent and then guess whether it was a human or a virtual player. Future work will also include looking into the joint improvisation among multiple human participants [28].

Finally we wish to highlight that the work presented in this paper opens the exciting possibility of performing *in-silico* experiments to assess how two human players (whose IMS have been recorded during solo trials) would interact when playing the mirror game in a JI condition. This can be useful for rehabilitation purposes such as those being explored as part of the EU project *AlterEgo* [29].

## Supporting Information

S1 File Parameter setting of the coupling weights for all the dyads and description of the available data. We show and interpret the values of *θ*_*p*,*i*_, *θ*_*σ*,*i*_ and *θ*_*v*,*i*_ for al the dyads of VPs used to test matching performance of the proposed mathematical model with the human benchmark. Moreover, we give a detailed description of all the available data and present tables showing the composition of the dyads mentioned in our analysis.
(PDF)

S1 Appendix Theoretical analysis of the proposed mathematical model of joint improvisation. We show that the solution to the minimization problem guarantees bounded position error between the two players involved in the game.
(PDF)

S1 Dataset S1_Dataset.mat. Matlab structure containing data from the interaction between two human players (HP-HP) recorded using experimental set-up 1.
(MAT)

S2 Dataset S2_Dataset.mat. Matlab structure containing data from simulated interactions between two virtual players (VP-VP).
(MAT)

S3 Dataset S3_Dataset.mat. Matlab structure containing data from the interaction between human and virtual players (HP-VP) recorded using experimental set-up 2.
(MAT)
