# Supplementary material for: Design of a Virtual Player for Joint Improvisation with Humans in the Mirror Game
Source: PLoS One. 2016 Apr 28;11(4):e0154361. doi: 10.1371/journal.pone.0154361 (PMC4849738; doi:10.1371/journal.pone.0154361)
Supplement: S1 File — We show and interpret the values of θp,i, θσ,i and θv,i for al the dyads of VPs used to test matching performance of the proposed mathematical model with the human benchmark. Moreover, we give a detailed description of all the available data and present tables showing the composition of the dyads mentioned in our analysis. (PDF) [file pone.0154361.s001.pdf]

S1 File

Chao Zhai [1], Francesco Alderisio [1], Piotr Słowiński [2], Krasimira Tsaneva-Atanasova [2], Mario di Bernardo [1,3,*]

1 Department of Engineering Mathematics, University of Bristol, BS8 1UB Bristol, United Kingdom
2 College of Engineering, Mathematics and Physical Sciences, University of Exeter, EX4 4QF Exeter, United Kingdom
3 Department of Electrical Engineering and Information Technology, University of Naples Federico II, 80125 Naples, Italy

*** Email: m.dibernardo@bristol.ac.uk**

A Parameter setting of the coupling weights for all the dyads

We here comment on the interpretation of the weights $\theta_{p,i}$, $\theta_{\sigma,i}$ and $\theta_{v,i}$, $i \in \{1,2\}$ for each dyad of VPs (Table A). These weights were tuned by trial-and-error according to the analysis of HP-HP interactions so that the VP dyads could achieve the desired matching results with the human benchmark.

Since the weights can be interpreted according to our theoretical model, they provide further insights onto the JI interaction between HPs. Table A shows that, in order for the model to replicate some characteristics of the human JI interaction in dyads 1, 2, 3, 6, 7 and 8, the weights had to belong to the following intervals:

Table A. Parameter setting of the coupling weights $\theta_{p,i}$, $\theta_{\sigma,i}$ and $\theta_{v,i}$ for all dyads, with $\eta_1 = \eta_2 = 10^{-4}$

Dyad1	VP1	$\theta_{p,1} = 0.10$	$\theta_{\sigma,1} = 0.30$	$\theta_{v,1} = 0.60$
	VP2	$\theta_{p,2} = 0.10$	$\theta_{\sigma,2} = 0.55$	$\theta_{v,2} = 0.35$
Dyad2	VP1	$\theta_{p,1} = 0.10$	$\theta_{\sigma,1} = 0.35$	$\theta_{v,1} = 0.55$
	VP2	$\theta_{p,2} = 0.12$	$\theta_{\sigma,2} = 0.45$	$\theta_{v,2} = 0.43$
Dyad3	VP1	$\theta_{p,1} = 0.15$	$\theta_{\sigma,1} = 0.30$	$\theta_{v,1} = 0.55$
	VP2	$\theta_{p,2} = 0.10$	$\theta_{\sigma,2} = 0.35$	$\theta_{v,2} = 0.55$
Dyad4	VP1	$\theta_{p,1} = 0.31$	$\theta_{\sigma,1} = 0.38$	$\theta_{v,1} = 0.31$
	VP2	$\theta_{p,2} = 0.31$	$\theta_{\sigma,2} = 0.38$	$\theta_{v,2} = 0.31$
Dyad5	VP1	$\theta_{p,1} = 0.72$	$\theta_{\sigma,1} = 0.22$	$\theta_{v,1} = 0.06$
	VP2	$\theta_{p,2} = 0.72$	$\theta_{\sigma,2} = 0.22$	$\theta_{v,2} = 0.06$
Dyad6	VP1	$\theta_{p,1} = 0.10$	$\theta_{\sigma,1} = 0.60$	$\theta_{v,1} = 0.30$
	VP2	$\theta_{p,2} = 0.10$	$\theta_{\sigma,2} = 0.28$	$\theta_{v,2} = 0.62$
Dyad7	VP1	$\theta_{p,1} = 0.10$	$\theta_{\sigma,1} = 0.30$	$\theta_{v,1} = 0.60$
	VP2	$\theta_{p,2} = 0.10$	$\theta_{\sigma,2} = 0.35$	$\theta_{v,2} = 0.55$
Dyad8	VP1	$\theta_{p,1} = 0.10$	$\theta_{\sigma,1} = 0.28$	$\theta_{v,1} = 0.62$
	VP2	$\theta_{p,2} = 0.10$	$\theta_{\sigma,2} = 0.30$	$\theta_{v,2} = 0.60$

- $\theta_{p,i} \in [0.10, 0.15], i \in \{1, 2\}$;
- $\theta_{\sigma,i} \in [0.28, 0.60], i \in \{1, 2\}$;
- $\theta_{v,i} \in [0.30, 0.60], i \in \{1, 2\}$.

This indicates that the corresponding HPs paid more attention to individual preferences (their IMSs) and mutual imitation than to position mismatches. On the other hand:

- $\theta_{p,4} = 0.31$, $\theta_{\sigma,4} = 0.38$, $\theta_{v,4} = 0.31$ lead to the conclusion that the corresponding HPs in Dyad 4 balanced all the three weights;
- $\theta_{p,5} = 0.72$, $\theta_{\sigma,5} = 0.22$, $\theta_{v,5} = 0.06$ lead to the conclusion that the corresponding HPs in Dyad 5 paid more attention to position error than to individual preferences and mutual imitation during their interaction.

B Available data

- **HP-HP.** Data was collected using experimental set-up 1. Data was recorded from 8 dyads (16 participants in total). Each recording has length 60sec. Sampling rate of the data is uniform and equal to 100Hz. Available data is saved as a Matlab (R2015b) .mat file.

  The file HPHP.mat contains a structure (HPHP) with 8 dyads fields (HPHP.dyad(i), i=1:8) each containing two players fields (HPHP.dyad(.).player(i), i=1:2). Each player field contains: field solo ((HPHP.dyad(.).player(.).solo) which is a 3x6000 matrix containing position traces from solo recordings, and field JI ((HPHP.dyad(.).player(.).JI) which is a 3x6000 matrix containing position traces from JI trials. Rows of the JI matrix of the two players in each dyad correspond to the three JI trials that they played together, e.g. data of the first JI trial for dyad 1 is contained in: HPHP.dyad(1).player(1).JI(1,:) and HPHP.dyad(1).player(2).JI(1,:).

  Corresponding time stamps can be generated in Matlab with the following command *t=0:0.01:59.99*.

- **VP-VP.** Simulations of the interactions between virtual players were run on a desktop computer using Matlab, source code available upon request. Each recording has length 60sec. Sampling rate is not constant due to changes in duration of simulation steps. In order to have uniform sampling rate, data in the paper is interpolated using mean time-step and shape-preserving piecewise cubic interpolation. Available data is saved as a Matlab (R2015b) .mat file.

  The file VPVP.mat contains a structure (VPVP) with 8 dyads fields (VPVP.dyad(i), i=1:8) each containing two players fields (VPVP.dyad(.).player(i), i=1:2). Each player field contains nine trials ((VPVP.dyad(.).player(.).trial(i), i=1:9) with field t (VPVP.dyad(.).player(.).trial(.).t) containing time stamps, and x (VPVP.dyad(.).player(.).trial(.).x) containing position traces from the simulated JI trials between two VPs. Additionally, each trial field contains field sig (VPVP.dyad(.).player(.).trial(.).sig) indicating which human solo recording was used as a reference signature in the simulations. For example, VPVP.dyad(1).player(1).trial(4).sig=2 indicates that in fourth simulation of dyad 1, player one was using the velocity from the second solo recording of human player 1 from dyad 1 as a signature. The position trace of that recording can be found in the HPHP.mat file in the second row of the solo matrix of dyad 1, player 1, i.e. HPHP.dyad(1).player(1).solo(2,:). Velocity traces corresponding to the position traces were computed using 4th order finite difference scheme.

Note that the 9 simulated trials correspond to all the possible combinations of the three solo recording of each of the two HPs who played together as a dyad in the HP-HP experiment. Position traces of the simulated trials of two virtual players playing together have the same trial number, e.g. data recorded in 6th simulation of dyad 4 can be found in VPVP.dyad(4).player(1).trial(6) and VPVP.dyad(4).player(2).trial(6).

- **HP-VP.** Data was collected using experimental set-up 2. Data was recorded for a single human participant interacting with a virtual player driven by a single motor signature. Each time series has length 60sec. Sampling rate is not constant due to changes in duration of time-steps of the experiment. Available data is saved as .mat Matlab file. HPVP.mat file contains HPVP data structure with following fields:

  1. HPVP.SigVP.x : position trace of a signature solo trajectory of human player recorded using leap motion (especially with the aim of the simulation); HPVP.SigVP.t : time stamps corresponding to position trace; HPVP.SigVP.v : velocity trace obtained from HPVP.SigVP.x by means of 1st order finite difference scheme. This velocity time-series has been used as an input (signature) for the interactive cognitive architecture driving the virtual player.
  2. HPVP.SigHP.x : position trace of a solo trajectory of human player recorded using leap motion. Position trace used as a signature of the VP, that can be found in HPVP.SigVP.x, comes from a different human player; HPVP.SigHP.t : time stamps corresponding to position trace; HPVP.SigHP.v : velocity trace obtained from HPVP.SigHP.x by means of 1st order finite difference scheme.
  3. HPVP.JIPosVP.x : position trace of the virtual player recorded during the JI trail; HPVP.JIPosVP.t : time stamps corresponding to position trace.
  4. HPVP.JIPosHP.x : position trace of the human player recorded during the JI trail; HPVP.JIPosHP.t : time stamps corresponding to position trace (they are the same as HPVP.JIPosVP.t).

Table B. Composition of dyads of VPs

Dyad	Trial	Sig. VP_1	Sig. VP_2
1–8	1	1	1
	2	1	2
	3	1	3
	4	2	1
	5	2	2
	6	2	3
	7	3	1
	8	3	2
	9	3	3

Table C. Data structure of a HP dyad

Dyad	Player	Condition	Matrix 3x6000	
1–8	1	Solo	1	$x1_{s1}$
			2	$x1_{s2}$
			3	$x1_{s3}$
		JI	1	$x1_{JI1}$
			2	$x1_{JI2}$
			3	$x1_{JI3}$
	2	Solo	1	$x2_{s1}$
			2	$x2_{s2}$
			3	$x2_{s3}$
		JI	1	$x2_{JI1}$
			2	$x2_{JI2}$
			3	$x2_{JI3}$

Table D. Data structure of a VP dyad

Dyad	Player	Trial	Fields: Signature, t, x		
1–8	1	1	1	t1	$x1_{JI1}$
		2	1	t2	$x1_{JI2}$
		3	1	t3	$x1_{JI3}$
		4	2	t4	$x1_{JI4}$
		5	2	t5	$x1_{JI5}$
		6	2	t6	$x1_{JI6}$
		7	3	t7	$x1_{JI7}$
		8	3	t8	$x1_{JI8}$
		9	3	t9	$x1_{JI9}$
	2	1	1	t1	$x2_{JI1}$
		2	2	t2	$x2_{JI2}$
		3	3	t3	$x2_{JI3}$
		4	1	t4	$x2_{JI4}$
		5	2	t5	$x2_{JI5}$
		6	3	t6	$x2_{JI6}$
		7	1	t7	$x2_{JI7}$
		8	2	t8	$x2_{JI8}$
		9	3	t9	$x2_{JI9}$
