# Supplementary material for: Design of a Virtual Player for Joint Improvisation with Humans in the Mirror Game
Source: PLoS One. 2016 Apr 28;11(4):e0154361. doi: 10.1371/journal.pone.0154361 (PMC4849738; doi:10.1371/journal.pone.0154361)
Supplement: S1 Appendix — We show that the solution to the minimization problem guarantees bounded position error between the two players involved in the game. (PDF) [file pone.0154361.s002.pdf]

S1 Appendix

Chao Zhai [1], Francesco Alderisio [1], Piotr Słowiński [2], Krasimira Tsaneva-Atanasova [2], Mario di Bernardo [1,3,*]

1 Department of Engineering Mathematics, University of Bristol, BS8 1UB Bristol, United Kingdom
2 College of Engineering, Mathematics and Physical Sciences, University of Exeter, EX4 4QF Exeter, United Kingdom
3 Department of Electrical Engineering and Information Technology, University of Naples Federico II, 80125 Naples, Italy

*** Email: m.dibernardo@bristol.ac.uk**

Theoretical analysis

We present here the theoretical analysis of the mathematical model we propose. More specifically we show that the solution to the minimization problem guarantees boundedness of the position error between the players when either two coupled VPs are considered or when the model-driven avatar interacts with a human subject.

Model-driven avatar

Let us recall that, over each sampling period $T = t_{k+1} - t_k$, the control input u is obtained by solving the optimal control problem:

$$\min_{u \in R} J \tag{1}$$

where

$$\begin{aligned} J = \frac{\theta_p}{2} \underbrace{(x(t_{k+1}) - \hat{r}_p(t_{k+1}))^2}_{Position\ TC} + \frac{\theta_\sigma}{2} \int_{t_k}^{t_{k+1}} \underbrace{(\dot{x}(\tau) - \sigma(\tau))^2}_{Motor\ Signature} d\tau \\ + \frac{\theta_v}{2} \int_{t_k}^{t_{k+1}} \underbrace{(\dot{x}(\tau) - \hat{r}_v(\tau))^2}_{Velocity\ TC} d\tau + \frac{\eta}{2} \int_{t_k}^{t_{k+1}} u(\tau)^2 d\tau \end{aligned} \tag{2}$$

with $\theta_p, \theta_\sigma, \theta_v, \eta > 0$ being tunable control parameters satisfying the constraint $\theta_p + \theta_\sigma + \theta_v = 1$. Here, x and $\dot{x}$ refer to position and velocity of the VP whose dynamics is modelled by:

$$\ddot{x} + (\alpha \dot{x}^2 + \beta x^2 - \gamma)\dot{x} + \omega^2 x = u \tag{3}$$

Moreover, σ refers to the IMS of a given HP in solo trials, while $\hat{r}_p$ and $\hat{r}_v$ represent the estimated position and velocity of the HP the VP is interacting with, respectively. In particular:

$$\hat{r}_v(t) = \frac{r_p(t_k) - r_p(t_{k-1})}{T} \quad t \in [t_k, t_{k+1}]$$

and

$$\hat{r}_p(t) = r_p(t_k) + \hat{r}_v(t)(t - t_k), \quad t \in [t_k, t_{k+1}]$$

Theorem 1. *The solution to the optimization problem described in Eq (1) ensures bounded position error between VP and HP.*

Proof. Let J_0 denote the value of the cost function described in Eq (2) when $u \equiv 0$. In addition, let J^* and x^* correspond to the optimal value of the cost function and the optimal position of the VP, respectively. It is clear that $J^* \leq J_0$ since J^* is the minimum value of the cost function. According to Theorem 5.1 in [1], there exists a limit cycle in the HKB oscillator, and thus x and $\dot{x}$ are bounded in J_0. Considering that $\hat{r}_p$, σ and $\hat{r}_v$ are all bounded, we conclude that J_0 is bounded. It follows from the inequality

$$\frac{\theta_p}{2}(x^*(t_{k+1}) - \hat{r}_p(t_{k+1}))^2 \leq J^* \leq J_0$$

that the position error between VP and HP is bounded as well. □

Corollary 1. *If the nonlinear HKB dynamics of the VP end-effector is substituted with a simpler linear dynamics of the form*

$$\ddot{x} + a\dot{x} + bx = u$$

achievement of the optimal solution to the minimization problem described in Eq (1) is guaranteed over each subinterval.

Proof. According to the fundamental theorem of the calculus of variations, we need to examine the second variation of the given cost function in order to establish the optimum. From existing conclusions in [2], the second variation of the cost function described in Eq (2) in the Hamiltonian formalism is given by

$$\delta^2 J = \theta_p[\delta x(t_{k+1})]^2 + \int_{t_k}^{t_{k+1}} \begin{pmatrix} \delta X & \delta u \end{pmatrix} \begin{pmatrix} H_{XX} & H_{Xu} \\ H_{uX}^T & H_{uu} \end{pmatrix} \begin{pmatrix} \delta X \\ \delta u \end{pmatrix} dt$$

where $\delta X = [\delta x \ \delta\dot{x}]^T$ and H is the Hamiltonian

$$H(X, u, \lambda) = \frac{1}{2}\theta_\sigma(\dot{x} - \sigma)^2 + \frac{1}{2}\theta_v(\dot{x} - \hat{r}_v)^2 + \frac{1}{2}\eta u^2 + \lambda^T \begin{pmatrix} \dot{x} \\ -ay - bx + u \end{pmatrix}$$

with $X = [x \ \dot{x}]^T$ and $\lambda = [\lambda_1 \ \lambda_2]^T$. Rewriting the linear system in matrix form we obtain

$$\dot{X} = AX + Bu$$

where

$$A = \begin{pmatrix} 0 & 1 \\ -b & -a \end{pmatrix}, \quad B = \begin{pmatrix} 0 \\ 1 \end{pmatrix}$$

Let $X = X^* + \delta X$ and $u = u^* + \delta u$, where X^* and u^* denote the optimal state and optimal control, respectively. Since $\dot{X}^* = AX^* + Bu^*$, we get

$$\delta\dot{X} = A\delta X + B\delta u \quad (4)$$

Thus, it follows from $H_{Xu} = H_{uX} = [0 \ 0]^T$, $H_{uu} = \eta > 0$ and

$$H_{XX} = \begin{pmatrix} 0 & 0 \\ 0 & \theta_\sigma + \theta_v \end{pmatrix} \geq 0$$

that

$$\begin{aligned}\delta^2 J &= \theta_p[\delta x(t_{k+1})]^2 \\ &\quad + \int_{t_k}^{t_{k+1}} \delta X(t)^T H_{XX} \delta X(t) + \eta(\delta u(t))^2 dt \\ &= \theta_p[\delta x(t_{k+1})]^2 \\ &\quad + \int_{t_k}^{t_{k+1}} (\theta_\sigma + \theta_v)(\delta \dot{x}(t))^2 + \eta(\delta u(t))^2 dt \\ &\geq 0\end{aligned}$$

Moreover, $\delta^2 J = 0$ is equivalent to $\delta x(t_{k+1}) = 0$, $\delta \dot{x}(t) = 0$ and $\delta u(t) = 0$ for all $t \in [t_k, t_{k+1}]$, which yields $\delta x(t) = \delta x(t_k) = 0$ from Eq (4). This corresponds to the optimal solution X^* and the optimal control u^*. Therefore, we conclude that the optimal control ensures achievement of the minimum value of the cost function described in Eq (2) over each sampling period. □

Two coupled VPs

Let us recall that the model of two interacting VPs we propose consists of two coupled HKB oscillators:

$$\begin{cases} \ddot{x}_1 + (\alpha_1 \dot{x}_1^2 + \beta_1 x_1^2 - \gamma_1)\dot{x}_1 + \omega_1^2 x_1 = u_1 \\ \ddot{x}_2 + (\alpha_2 \dot{x}_2^2 + \beta_2 x_2^2 - \gamma_2)\dot{x}_2 + \omega_2^2 x_2 = u_2 \end{cases} \tag{5}$$

where x_1 and x_2 refer to the positions of the two virtual players VP_1 and VP_2, respectively. Analogously to the previous case, the control input for each HKB oscillator can be derived by making each VP solve the following optimal control problem

$$\min_{u_i \in R} J_i, \quad i \in \{1, 2\}$$

where

$$\begin{aligned} J_1 &= \frac{\theta_{p,1}}{2}(x_1(t_{k+1}) - x_2(t_{k+1}))^2 \\ &\quad + \frac{\theta_{\sigma,1}}{2}\int_{t_k}^{t_{k+1}} (\dot{x}_1(\tau) - \sigma_1(\tau))^2 d\tau \\ &\quad + \frac{\theta_{v,1}}{2}\int_{t_k}^{t_{k+1}} (\dot{x}_1(\tau) - \dot{x}_2(\tau))^2 d\tau + \frac{\eta_1}{2}\int_{t_k}^{t_{k+1}} u_1(\tau)^2 d\tau \end{aligned}$$

and

$$\begin{aligned} J_2 &= \frac{\theta_{p,2}}{2}(x_2(t_{k+1}) - x_1(t_{k+1}))^2 \\ &\quad + \frac{\theta_{\sigma,2}}{2}\int_{t_k}^{t_{k+1}} (\dot{x}_2(\tau) - \sigma_2(\tau))^2 d\tau \\ &\quad + \frac{\theta_{v,2}}{2}\int_{t_k}^{t_{k+1}} (\dot{x}_2(\tau) - \dot{x}_1(\tau))^2 d\tau + \frac{\eta_2}{2}\int_{t_k}^{t_{k+1}} u_2(\tau)^2 d\tau \end{aligned}$$

with $\theta_{p,i}, \theta_{\sigma,i}, \theta_{v,i}, \eta_i > 0, i \in \{1, 2\}$ being tunable parameters satisfying the constraints $\theta_{p,i} + \theta_{\sigma,i} + \theta_{v,i} = 1$.

In order to perform theoretical analysis for the nonlinearly coupled system described in Eq (5), we formulate the Hamiltonian for each of the two previous optimization

problems as follows

$$H(X_i,u_i,\lambda_i)=\frac{1}{2}\theta_{\sigma,i}(\dot{x}_i-\sigma_i)^2+\frac{1}{2}\theta_{v,i}(\dot{x}_1-\dot{x}_2)^2+\frac{1}{2}\eta_i u_i^2 \\ +\lambda_i^T\begin{pmatrix}\dot{x}_i\\ -(\alpha_i x_i^2+\beta_i\dot{x}_i^2-\gamma_i)\dot{x}_i-\omega_i^2x_i+u_i\end{pmatrix}$$

where $X_i=[x_i\ \dot{x}_i]^T$ and $\lambda_i=[\lambda_{i1}\ \lambda_{i2}]^T$, $i\in\{1,2\}$. Applying the minimum principle [2], we get the optimal open loop control inputs given by

$$u_i=argmin_{u_i\in R}H(X_i,u_i,\lambda_i)=-\eta_i^{-1}\lambda_i^T\begin{pmatrix}0\\1\end{pmatrix}=-\eta_i^{-1}\lambda_{i2}$$

and the corresponding optimal state equations

$$\dot{X}_i=\nabla_{\lambda_i}H(X_i,u_i,\lambda_i) \\ =\begin{pmatrix}\dot{x}_i\\ -(\alpha_i x_i^2+\beta_i\dot{x}_i^2-\gamma_i)\dot{x}_i-\omega_i^2x_i-\eta_i^{-1}\lambda_{i2}\end{pmatrix} \tag{6}$$

with initial condition $X_i(t_k)=[x_i(t_k)\ \dot{x}_i(t_k)]^T$. Also, the optimal costate equations can be written as

$$\dot{\lambda}_1=-\nabla_{X_1}H(X_1,u_1,\lambda_1) \tag{7}$$

or equivalently as

$$\begin{cases}\dot{\lambda}_{11} & =\lambda_{12}(2\alpha_1x_1\dot{x}_1+\omega_1^2)\\ \dot{\lambda}_{12} & =\lambda_{12}(\alpha_1x_1^2+3\beta_1\dot{x}_1^2-\gamma_1)-\lambda_{11}-\theta_{\sigma,1}(\dot{x}_1-\sigma_1)\\ & -\theta_{v,1}(\dot{x}_1-\dot{x}_2)\end{cases}$$

and

$$\dot{\lambda}_2=-\nabla_{X_2}H(X_2,u_2,\lambda_2) \tag{8}$$

or equivalently

$$\begin{cases}\dot{\lambda}_{21} & =\lambda_{22}(2\alpha_2x_2\dot{x}_2+\omega_2^2)\\ \dot{\lambda}_{22} & =\lambda_{22}(\alpha_2x_2^2+3\beta_2\dot{x}_2^2-\gamma_2)-\lambda_{21}-\theta_{\sigma,2}(\dot{x}_2-\sigma_2)\\ & -\theta_{v,2}(\dot{x}_2-\dot{x}_1)\end{cases}$$

with terminal conditions

$$\lambda_1(t_{k+1})=\begin{pmatrix}\theta_{p,1}(x_1(t_{k+1})-x_2(t_{k+1}))\\0\end{pmatrix}$$

and

$$\lambda_2(t_{k+1})=\begin{pmatrix}\theta_{p,2}(x_2(t_{k+1})-x_1(t_{k+1}))\\0\end{pmatrix}$$

Hence, the solution of the coupled model can be obtained by solving the above boundary value problem described by Eq (6), Eq (7) and Eq (8). Following the same proof strategy as in Theorem 1, it is guaranteed that the position error between two VPs is bounded, and the proof is thus omitted to avoid redundancies. In particular, the achievement of joint improvisation movement is available by properly tuning the parameters $\theta_{p,i}$, $\theta_{\sigma,i}$ and $\theta_{v,i}$.

References

1. Zhai C, Alderisio F, Tsaneva-Atanasova K, di Bernardo M. A model predictive approach to control the motion of a virtual player in the mirror game. In: 54th IEEE Conference on Decision and Control (CDC). IEEE; 2015. p. 3175–3180.

2. Naidu DS. Optimal control systems. CRC press; 2002.
